# Family physicians’ views on their role in the management of childhood obesity: a mixed methods study from Turkey

**DOI:** 10.1080/13814788.2018.1503247

**Published:** 2018-09-26

**Authors:** Sibel Sakarya, Pemra C. Ünalan, Naz Tursun, Anıl Özen, Seda Kul, Ümit Gültekin

**Affiliations:** a Medical Faculty, Public Health Department, Marmara University, Istanbul, Turkey;; b Medical Faculty, Family Medicine Department, Marmara University, Istanbul, Turkey;; c Medical Faculty, Marmara University, Istanbul, Turkey

**Keywords:** Child obesity, mixed methods, parents, primary care physicians, Turkey

## Abstract

**Background:** Childhood obesity (CO) is a high priority issue due to its serious health consequences and its rapid increase.

**Objectives:** To examine the views of primary care physicians (family physicians, FPs) in Turkey regarding their role in the management of CO and the barriers they perceive.

**Methods:** Mixed methods approach. Data was collected in two major counties of Istanbul between February and May 2014. All Family Health Centres (FHCs) in the region were visited, and 180/284 FPs (63.4%) agreed to complete a structured questionnaire (22 questions). Of those, 48 FPs agreed to participate in in-depth interviews that were taken until saturation was 25. Quantitative data were analysed using descriptive statistics. For qualitative data, content analysis was applied to identify the themes.

**Results:** Most of the FPs (93.3%) agreed that they have a role in managing CO. Almost all FPs (98.3%) agreed that for the 0–4-year-olds height and weight measures should be taken. However, only 67.6% recommended this for children aged 5–15 years. The most common barriers in the management of CO were reported as lack of time (68.9%) and FHCs not being utilized for the care of children aged 5–15 years old (53.3%) in Turkey. In-depth interviews showed that FPs tend to limit their role to identifying the problem and making the family aware of it.

**Conclusion:** Although FPs recognize primary healthcare as an appropriate setting for managing CO, they have concerns about being involved in the treatment.

KEY MESSAGES

- Family physicians (FPs) recognize primary healthcare as an appropriate setting for managing childhood obesity (CO).
- Most reported barriers in the management of CO were lack of time and the fact that Family Health Centres are not being utilized regularly for the care of children aged 5–15 years in Turkey.
- FPs tend to limit their role in identifying the problem and making the family aware of it.

## Introduction

Childhood obesity (CO) is considered one of the most serious public health challenges of the early twenty-first century due to its rapid increase and severe health consequences [1]. Although the problem is global, it mainly affects low and middle-income countries and urban areas. There are approximately 42 million overweight children in the world in 2013 and close to 31 million of these live in developing countries [2].

The problem has a high priority in Turkey. Today, obesity is accepted as one of the most frequently seen chronic diseases among children [3]. Many regional and several national surveys have been conducted so far to define the dimensions of the problem, and several strategy documents have been prepared for the prevention and management of CO [3–5]. In Turkey in 2009, the prevalence of overweight in 6–9-year-old children was found to be 14.3% and of obesity 6.5% [4]. In another study carried out in 2013 among 7 and 8-year-old children, the prevalence of overweight was 14.2% and of obesity was 8.3% [5].

It is essential to recognize obesity at an early stage and treat it appropriately to prevent it. In Turkey, Family Health Centres (FHCs) are responsible for individual-based prevention and treatment. In these centres no other specialists, but only general practitioners and family medicine specialists, are working and are all named as family physicians (FPs). They provide primary healthcare services for all age groups. Even though the primary care services are identified as the main source responsible for managing CO, there are various barriers for the effective management of the problem identified in the literature. Lack of training, time constraint, lack of resources, concerns related to communicating with the parents about child’s weight, health system related factors such as lack of reward were identified as important barriers to addressing childhood obesity [6–13].

Almost all CO research in Turkey has focused on epidemiological and clinical aspects of the problem, and there are no studies which allow determining the factors impeding the diagnosis, treatment, and follow-up of CO in primary care. We planned our study to examine the views of FPs about their role and the barriers they perceive in the management of CO.

## Methods

### Study design

We used a mixed methods approach in which both quantitative and qualitative methods were employed [14]. The data were collected between February and May 2014 among FPs in two major counties of Istanbul. These counties were the oldest, well-settled, and central neighbourhoods of the city with residents of middle class and at least five years of school education. Quantitative data was collected with a structured questionnaire; in-depth interviews obtained qualitative data. The reason for adding qualitative data to the study was to elaborate the views of FPs about their roles in the management of CO and the barriers they perceived. During the in-depth interview, we asked them to tell us more about these issues.

## Ethics

Ethical approval was obtained from the Ethical Committee of Marmara University Institute of Health Sciences in 07.02.2014 (protocol no. 1400031936).

### Data collection—quantitative study

All 82 FHCs in the region were visited. Of 284 FPs assigned at these centres, 180 FPs volunteered to be complete a structured questionnaire (63.4%). The questionnaire consisted of 22 questions on demographic and occupational characteristics of the physicians and questions related to their attitude and practice on the management of CO in primary care. The questions were taken from a questionnaire used in a study carried out in Australia [12]. The questionnaires were distributed and recollected by the four researchers at FPs offices.

### Data collection—qualitative study

When the questionnaire had been completed, the physicians were asked if they would be willing to join a follow-up in-depth interview, and those who agreed (48 FPs) were asked for their contact information. We had in-depth interviews using a semi-structured interview guide until we reached saturation. We covered three issues in the guide: how do FPs perceive their role in the management of CO (including reasons behind), what are their explanations about the obstacles, and what do they suggest for solutions. For the enrolment to the in-depth interview, the diversity of the answers was prioritized, and the interviews were continued until saturation was achieved (after 25 interviews) [15]. The same four authors interviewed the FPs at their offices and recorded the interviews with the consent of the physicians. Interviews took approximately 20–25 min.

### Data analysis

Quantitative data were analysed using descriptive statistics. For the in-depth interviews, tape recordings were analysed and written verbatim the same day. The text was read and coded by two researchers independently. Content analysis was applied to identify the themes.

## Results

### Quantitative results

#### Participants

Table 1 shows the characteristics of the participating FPs. Mean age of the participating FPs was 43 years (SD = 7.0), and 46.8% of them were female. The mean of their work experience was 18 years (Table 1). The number of paediatric patients examined by FPs per week varied; the median was 30. A total of 24.4% of physicians had taken a postgraduate training course on CO.

**Table 1.** Demographic characteristics of the family physicians who completed the quantitative questionnaire (*n* = 180).

Gender: *n* (%)	
Male	96 (53.2)
Female	84 (46.8)
Type of FPs: *n* (%)	
FP specialists	35 (20.0)
General practitioners	145 (80.0)
Mean age ± SD (min-max)	43.0 ± 7.0 (26–63 years)
Experience in general practice	Range

FP, family physician.

#### Responsibility

Asked for their opinion on who is responsible for tackling CO, 93.3% (*n* = 168) of the physicians answered affirmatively to the question ‘Is there any role for FPs in managing childhood obesity?’ (10 physicians replied ‘no,’ and two did not answer). According to the physicians, the most important roles in managing CO belong to the parents (91.6%), primary care physicians (74.3%) and teachers (52.5%). The role of the paediatricians is similar to that of the media (28.5% and 26.4%, respectively) (Table 2).

**Table 2.** Family physicians’ opinions about the main person in charge of the childhood obesity management (*n* = 179)^a^.

	*n*	%
Parents	164	91.6
Primary care physician (FP)	133	74.3
School and the teacher	94	52.5
Paediatrician	51	28.5
Media	47	26.3
Dietitian	17	9.5
Health authority (Ministry of Health, etc.)	15	8.4
The child itself	14	7.8

^a^One FP did not answer. A participant can sign more than one item. Percentages were calculated based on number of participants (*n* = 179).

FP, family physician.

#### Attitude and behaviour of FPs

Although 98.3% of physicians agree that it is necessary to evaluate measurements of height and weight of children in their first four years, this percentage dropped to 67.6% for the 5–15 year of age group (Table 3). The percentage of FPs who provides advice to an overweight child and his family is 66.1%. Of the FPs 47.5% (*n* = 84) feel self-reliant to manage children who are overweight or obese; while a half (46.7%) suggest that the best choice to refer these children to other professionals rather than attempting to treat them (Table 3).

**Table 3.** Attitudes and behaviours of the family physicians about childhood obesity management (*n* = 180)^a^.

Opinions	Agree		Disagree		Undecided	
	*n*	%	*n*	%	*n*	%
Measuring weight and height for the kids between 0 and 4 years of age is a requirement.	176	98.3	–	–	3	1.7
Measuring weight and height for the kids between 5 and 15 years of age is a requirement.	121	67.6	17	9.5	41	22.9
I can give consultancy to an overweight or obese child and his family.	119	66.1	19	10.6	41	23.3
Consultancy to an overweight or obese child and his family is professionally a satisfaction for me.	117	65.7	21	11.7	40	22.6
I am professionally well prepared to work with children and families from diverse social and cultural backgrounds	101	56.1	22	12.3	56	31.6
It is difficult to understand that a child is overweight or obese just by looking at him.	92	51.7	65	36.5	21	11.8
I am professionally self-reliant to manage children who are overweight or obese	84	47.5	22	12.4	71	40.1
The best role for a GP is to refer overweight and obese children to other professionals rather than attempting to treat them.	84	46.7	75	42.1	19	11.2
Only a small percentage of children who are overweight/obese can reduce their BMI and maintain that loss for at least a year.	32	17.8	102	57.0	44	25.2
I would only encourage weight management when a child or his family request it	19	10.7	140	78.7	19	10.6
Weight measurement should be offered only to obese children.	15	8.4	151	83.9	13	7.7

^a^There are missing data for each item.

#### Barriers

The most common obstacle in CO management for the physicians is ‘lack of time’ (68.9%) and the absence of the 5–15 year age group visits to the FHCs in Turkey (53.3%) (Table 4).

**Table 4.** Barriers reported by the family physicians regarding the management of childhood obesity (*n* = 180)^a^.

	*n*	%
Lack of time	124	68.9
5–15 years of age groups visits to FHCs are not required	96	53.3
Lack of motivation of the FPs	65	36.1
Limited training/knowledge	55	30.5
Lack of the necessary tools	33	18.3

^a^A participant can report more than one barrier. Percentages were calculated based on number of participants (*n* = 180).

FPs, family physicians; FHCs, family health centres.

### Qualitative results

Among the 25 FPs (14 females) we interviewed, 20 were GPs, and five were FP specialists.

### Role of FPs in the management of childhood obesity

In the quantitative part of the study, a vast majority of the FPs had stated that FPs should have a role in the management of CO. During the in-depth interview, we asked 25 of them to tell us more about this role. We found that all of the participants tend to limit the role of FPs in identifying the problem and making the family aware of it. According to most of the FPs, this was due to the task definition of FPs; while a few of them said FPs would do further investigations if there were enough facilities and opportunities such as laboratory tests, etc.

… I mean … to detect obesity, to warn kids and the families … this is what they (FPs) can do. (FP No. 23)

… the tests are so limited over here, therefore we can only catch the problem. (FP No. 12)

### Barriers in the management of childhood obesity

In the second part of the interview, FPs were asked about perceived barriers and suggested solutions. The issues were evaluated in three main categories: healthcare system, parents, and physicians. These qualitative results are summarized in Table 5.

**Table 5.** Qualitative results: summary of opinions of family physicians about barriers for childhood obesity management in primary care (25 in-depth interviews).

Barriers for childhood obesity management in primary care		
Barriers related to the healthcare system	Barriers related to parents/ families	Barriers related to physicians
Excess workload of the primary healthcare services in Turkey The ‘treatment’ focus of current primary healthcare system FHCs are not being used for healthy child (5–15 years old) follow-ups Lack of integration between primary and secondary care	Positive attitude of parents towards overweight/obese child Knowledge deficiency of parents about healthy nutrition	Lack of knowledge and training regarding management of CO Lack of motivation

FHCs, family health centres; CO, childhood obesity.

### Barriers associated with the healthcare system

When asked about the difficulties, most of the FPs answered that it was the excessive workload of the primary healthcare services. The FPs pointed out not only the disproportionate number of patients but also the range of duties.

… here is a busy place because there is not only obesity but also other preventive services. There are also many prescriptions (to be repeated). Then follow-up of healthy children, vaccinations, pregnancies and elderly people. So I put time limitation to the top of the list. (FP No. 15)

Another problem mentioned related to the system was the lack of integration and of data sharing between primary and secondary care. FPs do not know when and where to refer to when they have such a patient; they complain about getting no feedback.

Families who have the patience to refer to a secondary care unit, their number is low, they don’t come back to me and share the results with me after 1–2 months. The child that we referred is lost there (FP No. 15)

FPs point out that they cannot see children older than five years for follow-ups unless they have an acute illness. For 5–15-years age group, routine follow-ups are mainly preferred to be done by the paediatricians. So FPs have fewer interactions with this age group to observe if they are obese or not.

In the primary care system we follow-up under five year-old children for height and weight, older than five years? … hmmmm. We do not… (FP No. 1)

### Barriers associated with parents

Parents’ positive attitude towards an overweight child is mentioned as a serious barrier by most of the physicians. According to FPs, for some parents’ perspective, an overweight child is widely accepted more popular than a thin child is.

… parents don’t visit me for childhood obesity. The child’s obesity is justifiable for the parents. They are even happy. (FP No. 11)

… Families tend to feed, to stuff the children from their early childhood. They compare the children with each other; if they think their child is thinner, they start force-feeding. For some parents, it is not sufficient to tell them three, four or even five times, to convince them of their child’s obesity. (FP No. 9)

### Barriers associated with physicians

Some FPs expressed that they had received no education and their lack of knowledge and skills were treated as barriers. Another group of FPs reported that they are sufficiently equipped with this subject but they do not have enough time and motivation. Some physicians mentioned the lack of clinical guidelines and documents about childhood obesity that would support them.

… we did not have an intense education about preventive health services during our education. It was mainly about the diseases and therapeutic medicine. (FP No. 1)

… In fact, they (FPs) are both well informed and equipped. However, I do not know if they spare the time… (FP No. 14)

### Recommendations for the management of childhood obesity

Recommendations of FPs are summarized in Table 5. They include utilization of the media for the parents and community education to empower the schools and teachers, to focus the primary healthcare system on preventive medicine, to improve the integration between primary and secondary healthcare system, to reduce the number of patients per physician and their workload.

… In addition to the physicians, there are three essentials in children’s education. Media! They need to watch on the screen that obesity is not normal. Family and education are important. If all these realize then physicians don’t need to put more effort in this subject. (FP No. 7)

… at schools, there should be special menus prepared for obese children, and teachers must tell the children that obesity is a disease. At this point, the responsibility of the schools and the teachers are much more important than the families and even more than us. (FP No. 20)

## Discussion

### Main findings

Using a mixed-methods approach, FPs opinions about their role in the management of CO in primary care setting were evaluated. Most FPs agreed that they have a role in managing CO. Almost all FPs agreed that for the 0–4-year-olds’ height and weight measures should be taken. However, only two thirds of FPs recommended this for children aged 5–15 years. The most reported barriers in the management of CO were lack of time and the fact that in Turkey FHCs are not being utilized for the care of children aged 5–15 years. In-depth interviews showed that FPs tend to limit their role to identifying the problem and making the family aware of it.

### Limited role of FPs

The critical role of the FPs and primary care setting in the management of obesity is well known [13, 16, 17]. In the current study, half of the FPs stated that ‘the main role of an FP is to refer obese children to other professionals rather than attempting to treat them.’ In a similar study in Australia, however, only 20% of the general practitioners agreed [12].

The results of the qualitative part of the study showed that there might be two reasons behind this opinion of FPs. First, FPs accept ‘prevention’ to be their primary role. Second, due to the lack of technical (i.e., specific laboratory tests) and professional support (lack of integration or coordination with other disciplines), they limit their role. Lack of integration or coordination between primary and secondary healthcare has been mentioned as a problem of primary healthcare in previous studies [11, 18–20].

### Barriers and solutions

#### Knowledge

Lack of knowledge regarding management of CO was also reported in this study as a barrier among FPs. This lack may indicate a need of effective training of FPs in the management of CO. All these factors were reported in other studies as factors discouraging FPs from treating CO [6, 9–11]. However, a lack of standardized guidelines was mentioned by only a few FPs in our study; although this has been reported as an important problem in other studies [11, 13, 21].

#### Motivation

Almost 40% of FPs in our study indicated a lack of motivation as a barrier for the management of CO. Weakness of the PHC system in Turkey seems to be a major barrier for this low motivation [19, 22, 23]. For instance, in Turkey there are no incentives for better performance such as CO management. However, payment cut off occurs if FPs cannot reach specific targets.

#### Workload

Lack of time and an excess workload of FPs were reported in both quantitative and qualitative parts of our study. Lack of time has been reported by FPs in Turkey as well as in other studies from different countries [10, 11, 13, 22]. In our research, FPs related these two issues with the set-up of the PHC system. The high number of patients per FPs and low rate of utilization of FHCs as the first contact care were addressed as the challenges of PHC system in Turkey [24].

#### Limited contact at school age

Another critical issue related to problems of PHC in Turkey that fails the role of primary care in the management of CO was that FHCs were not being utilized for child monitoring after school age. In different studies, practitioners also reported limited contact with children [11, 25, 26].

#### Responsibility

According to several previous studies, FPs think that primary care is the key place in the management of CO [7, 9–11, 20, 27]. Although we found a similar result in our study, FPs have given the maximum priority in the management of CO to the parents (91.6%); school and teachers (52.5%) following the FPs (74.3%) in third place. Parental participation and cooperation are key factors in the prevention and management of CO [28–32]. Parents’ lack of recognition or concern about obesity in children has been one of the main barriers in managing the CO [8, 10, 11, 13, 22, 33]. FPs in our study recommended better cooperation between school and parents and using the power of media to increase awareness in the society.

### Strengths and limitations

This is the first study in Turkey that used a mixed methods approach to understand FPs views and experiences about managing the CO at primary healthcare. The qualitative part of our mixed methodology design helped us elaborating answers to the closed-ended questions that were used in the structured questionnaire. Through interviews, we asked physicians to provide us with in-depth information about the role of the FPs in CO management, barriers about the subject and suggested solutions. This is the strength of our study. Both the low response rate of the study (63.4%) and volunteer participation of the FPs are the limitations of our study. Therefore, the results of the study might not reflect opinions of all the FPs in the two counties studied and the situation in other parts of the country.

The data for this study was collected in 2014. Because there has been no structural or organizational change in the primary healthcare regarding management of CO since that date, we do not consider this a significant limitation.

### Implications for clinical practice, policy and education

We found that FPs recognized primary healthcare as an appropriate setting for managing the CO while at the same time they are having concerns about involving in the treatment. Our study added new knowledge on perceived barriers to a stronger role of FPs and most of these were attributed to the primary healthcare system.

We recommend reducing the number of patients per FPs, to implement indicators for the follow-up of 5–15-year-old children in primary care system with specific guidelines, to provide effective in-service training for FPs, and to stimulate better collaboration between FPs and specialists from other disciplines.

## Conclusion

FPs recognize primary healthcare as an appropriate setting for managing CO but tend to limit their role in identifying the problem and making the family aware of it. Most reported barriers in the management of CO were ‘lack of time’ and Turkish FHCs not being utilized for the care of children aged 5–15 years.
